# Universal HbA1c Measurement in Early Pregnancy to Detect Type 2 Diabetes Reduces Ethnic Disparities in Antenatal Diabetes Screening: A Population-Based Observational Study

**DOI:** 10.1371/journal.pone.0156926

**Published:** 2016-06-07

**Authors:** R. C. E. Hughes, J. Williman, J. E. Gullam

**Affiliations:** 1 Department of Obstetrics and Gynaecology, University of Otago, Christchurch Women’s Hospital, Christchurch, New Zealand; 2 Biostatistics and Computational Biology Unit, University of Otago, Christchurch School of Medicine, Christchurch, New Zealand; East Tennessee State University, UNITED STATES

## Abstract

In response to the type 2 diabetes epidemic, measuring HbA1c with the first-antenatal blood screen was recently recommended in NZ. This would enable prompt treatment of women with unrecognised type 2 diabetes, who may otherwise go undetected until the gestational diabetes (GDM) screen. We compare inter-ethnic antenatal screening practices to examine whether the HbA1c test would be accessed by ethnicities most at risk of diabetes, and we determined the prevalence of unrecognised type 2 diabetes and prediabetes in our pregnant population. This is an observational study of pregnancies in Christchurch NZ during 2008–2010. Utilising electronic databases, we matched maternal characteristics to first-antenatal bloods, HbA1c, and GDM screens (glucose challenge tests and oral glucose tolerance tests). Overall uptake of the first-antenatal bloods versus GDM screening was 83.1% and 53.8% respectively in 11,580 pregnancies. GDM screening was lowest in Māori 39.3%, incidence proportion ratio (IPR) 0.77 (0.71, 0.84) compared with Europeans. By including HbA1c with the first-antenatal bloods, the number screened for diabetes increases by 28.5% in Europeans, 40.0% in Māori, 28.1% in Pacific People, and 26.7% in ‘Others’ (majority of Asian descent). The combined prevalence of unrecognised type 2 diabetes and prediabetes by NZ criteria, HbA1c ≥5.9% (41mmol/mol), was 2.1% in Europeans, Māori 4.7% IPR 2.59 (1.71, 3.93), Pacific People 9.5% IPR 4.76 (3.10, 7.30), and ‘Others’ 6.2% IPR 2.99 (2.19, 4.07). Applying these prevalence data to 2013 NZ national births data, routine antenatal HbA1c testing could have identified type 2 diabetes in 0.44% and prediabetes in 3.96% of women. Routine HbA1c measurement in early pregnancy is an ideal screening opportunity, particularly benefitting vulnerable groups, reducing ethnic disparities in antenatal diabetes screening. This approach is likely to have world-wide relevance and applicability. Further research is underway to establish whether, as for type 2 diabetes, prompt treatment of prediabetes improves pregnancy and neonatal outcomes.

## Introduction

Globally approximately half of all people with type 2 diabetes are undiagnosed and the age of onset is falling [1]. In response to this, the New Zealand Ministry of Health recommends that an HbA1c test be offered to all pregnant women at booking as part of the first antenatal blood screen to detect unrecognised type 2 diabetes [2]. HbA1c should ideally be tested before 20 weeks’ gestation in order to make the most of the opportunity to improve maternal and infant outcomes, and before glycaemic control deteriorates further with the onset of pregnancy-induced insulin resistance. To date, the majority of women with unrecognised type 2 diabetes were identified at the time of routine screening for gestational diabetes mellitus (GDM), by which time pregnancy complications may already be present. As with type 2 diabetes, recent evidence indicates that prediabetes is also associated with adverse pregnancy outcome, such as congenital anomalies, pre-eclampsia, spontaneous pre-term birth, and shoulder dystocia [3]. In addition, a NZ study reports that women with prediabetes who were not referred and treated until after 24 weeks gestation had worse pregnancy outcomes than women with a normal booking HbA1c who subsequently developed GDM [4]. It is established that prompt treatment of type 2 diabetes improves pregnancy outcomes, but data are lacking on the benefit of treating prediabetes in early pregnancy, although reduced rates of pre-eclampsia and spontaneous preterm birth are reported in women treated promptly versus those treated after 24 weeks gestation [4].

Universal HbA1c measurement at booking is also an opportunity to screen vulnerable groups with a low uptake of GDM screening. Despite a universal approach to GDM screening, reported rates of uptake in population-based studies are highly variable ranging from 50.6% to 91% [5–8], with lower screening rates seen in primary care settings and disparities in uptake by ethnicity and age. In NZ, indigenous Māori have a relative high prevalence of type 2 diabetes in children [9], yet the reported prevalence of GDM in Māori is only 3.3%, less than half the rate found in other non-European groups with similar rates of type 2 diabetes [2]. This may indicate an ethnic disparity in the uptake of antenatal diabetes screening as reported by others [5,6]. Māori and Pacific babies are more likely to be born prematurely, have higher rates of perinatal death, and higher rates of neonatal encephalopathy with evidence of asphyxia present at the time of birth [10]. Thus any steps in reducing ethnic disparities in the uptake of antenatal care are important to achieve.

The question remains, however, will universal HbA1c measurement at booking be accessed by those most at risk of unrecognised type 2 diabetes and prediabetes and can this approach reduce ethnic disparities in antenatal diabetes screening practices? Our aims were to examine inter-ethnic antenatal screening practices, including the uptake of both the first-antenatal blood screen and the universal two-step GDM screen recommended at 24–28 weeks gestation. Secondly, to compare the prevalence by ethnicity of unrecognised type 2 diabetes and prediabetes from HbA1c testing.

## Methods

This is an observational population-based retrospective cohort study examining data collected and entered into laboratory and the local District Health Board databases. Approval was granted by the Southern Health and Disability Ethics Committee of NZ, verbal informed consent was obtained by primary health care providers (family doctors and midwives) for the blood tests, no consent was required for the analysis as the data were de-identified. The ethics committee approved the verbal consent process as it was not obtained directly by the researchers and it is standard practice to discuss and obtain verbal consent for all antenatal tests. The HbA1c tests were subsequently ordered by family doctors and midwives with the researchers billed by the laboratories who had labelled the study bloods with a unique code. This consent process enabled more widespread HbA1c testing than could be obtained by the researchers alone. From 1^st^ February 2008 to 31^st^ August 2010, women in the Christchurch region were offered an HbA1c measurement with their routine first-antenatal blood screen in the primary care setting, as part of the ‘screening for diabetes in early pregnancy study’ [3]. During this former study, no lifestyle advice or treatment was offered to women with elevated HbA1c measurements, however they were counselled by their family doctor or midwife that they may have a higher risk of GDM and they were offered an early GDM screen (glucose tolerance test (GTT)) although the uptake of this was low [3]. We collected clinical data (including maternal ethnicity, age and estimated delivery date) for all births recorded in the local District Health Board database that had an estimated date of conception between 1^st^ February 2008 and 31^st^ January 2010 inclusive. These records were matched to laboratory data (date of test and results of HbA1c measurements, glucose challenge test (GCT), and GTT). Matching was conducted using the mother’s NHI number (a unique health identifier used in NZ), and ensuring that the date of test occurred between the estimated date of conception and date of delivery. We excluded women with known diabetes, no documented ethnicity, and those who lived outside the study area but who were transferred to Christchurch for the birth.

Three independent laboratories undertook blood sampling and analysis and all used identical analytical methods. HbA1c measurements were determined by Bio-Rad Variant 2 high performance liquid chromatography and if an abnormal haemoglobin variant was detected HbA1c was measured using the Bio-Rad In2It affinity chromatography radioimmunoassay. It was recognised that there may be up to a 0.02 inter-laboratory error at the range of HbA1c levels that we were interested in.

### Statistical Analysis

The proportion of women undertaking each of the screening tests at any time during pregnancy, and proportion exceeding test cut-off values, were summarised descriptively using counts and percentages, by ethnicity and age (categorised as five year bands). These incidence proportions (risks) were compared between ethnicities by calculating unadjusted and age-adjusted incidence proportion ratios (IPR) with 95% confidence intervals, using poisson regression models with “sandwich” standard errors [11]. Age was included in adjusted models as a continuous linear or quadratic variable. Socioeconomic status was not recorded in the data set thus we could not adjust for this in the analysis, however in New Zealand maternity care is free for all residents and eligible visa holders and is funded by taxation. Gestation at test was summarised descriptively using median and interquartile ranges. All analysis was performed using R 3.2.3 [12]. Regression models were fitted using Thomas Lumley’s *survey* package [13], and figures prepared using the *ggplot2* package [14].

## Results

During the 24-month study period, there were 11939 births of which 359 met our exclusion criteria. The remaining 11580 births in the final analysis group were to 11246 women, European 8706 (77.4%), Māori 931 (8.3%), Pacific People 401 (3.6%), and 1208 (10.7%) to ‘Others’ the majority of who were of Asian descent. There was marked inter-ethnic variability in maternal age at delivery, the median (inter-quartile range (IQR)) in European women was 31 years (26, 35), Māori 25 (21, 31), Pacific People 28 (23, 32), ‘Others’ 30 (27, 34), and further results are adjusted for maternal age.

**Fig 1:**
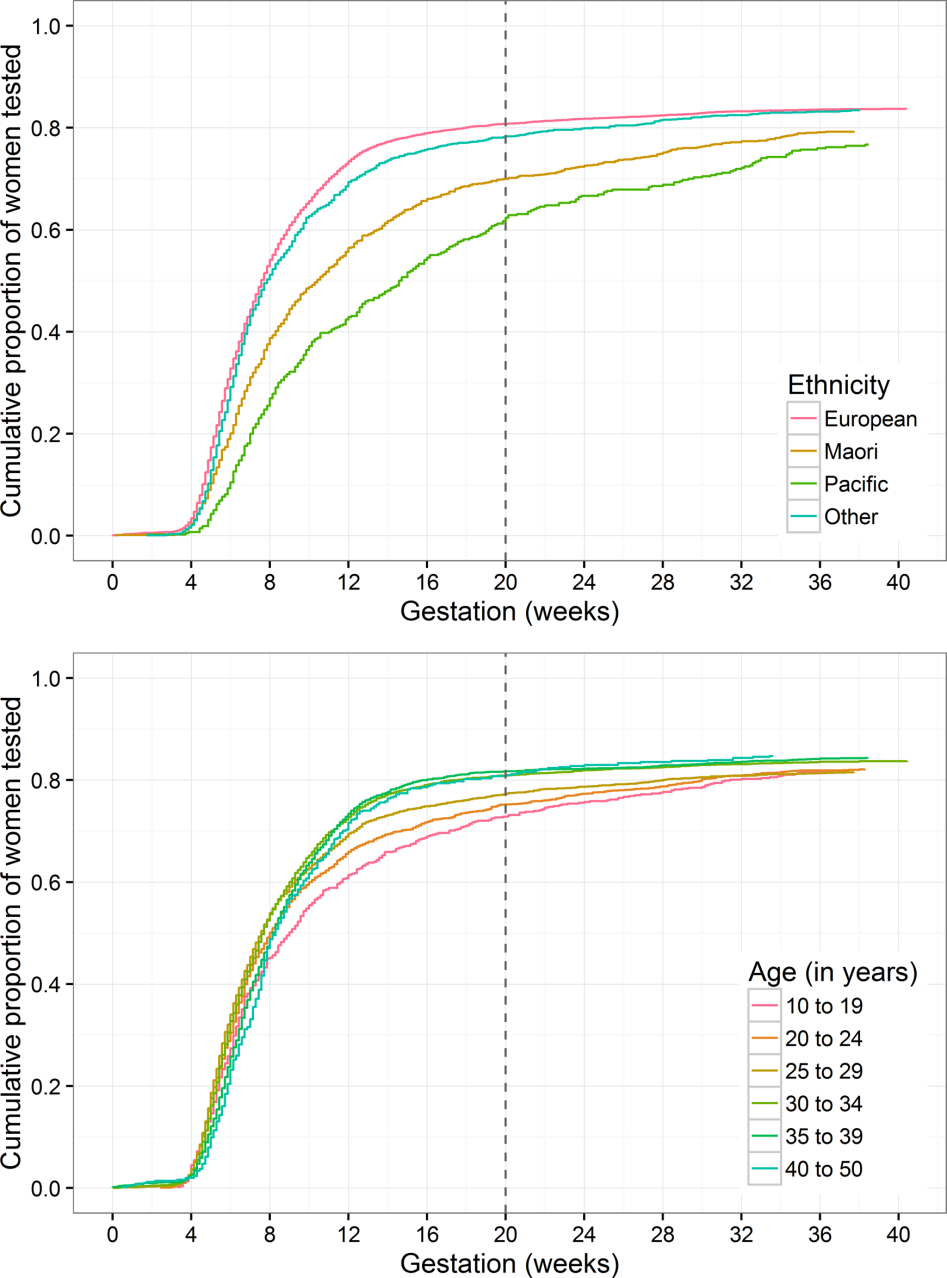
Cumulative Uptake of the First Antenatal Bloods by Weeks’ Gestation from Conception Through to Delivery. Fig 1a. Grouped by maternal ethnicity. Fig 1b. Grouped by maternal age.

The overall uptake of the first-antenatal bloods was 83.1%, with a slight approximately linear increase by age from 81.8% in under 20 year olds up to 84.9% in over 40 year olds (p = 0.037). Compared with European women, Māori and Pacific People were slightly less likely to be tested, adjusted (adj) IPR (95% confidence interval) 0.95 (0.92 to 0.99) and adj IPR 0.92 (0.87 to 0.97) respectively, while there was no difference in test uptake by ‘Others’ adj IPR 1.00 (0.97 to 1.03). The median (IQR) gestation in weeks at the time of the first-antenatal blood tests in European woman was 6.9 (5.3, 9.4), Māori 8.3 (60, 13.3), Pacific People 10.3 (7.0, 17.6), and ‘Others’ 7.0 (5.6, 10.0). Of those who had a first-antenatal blood test, Pacific Island women were most likely to be tested after 20 weeks’ gestation (20.5%, versus 11.4% Maori, 6.8% ‘Others’, and 3.2% European, p<0.001) Fig 1.

**Fig 2:**
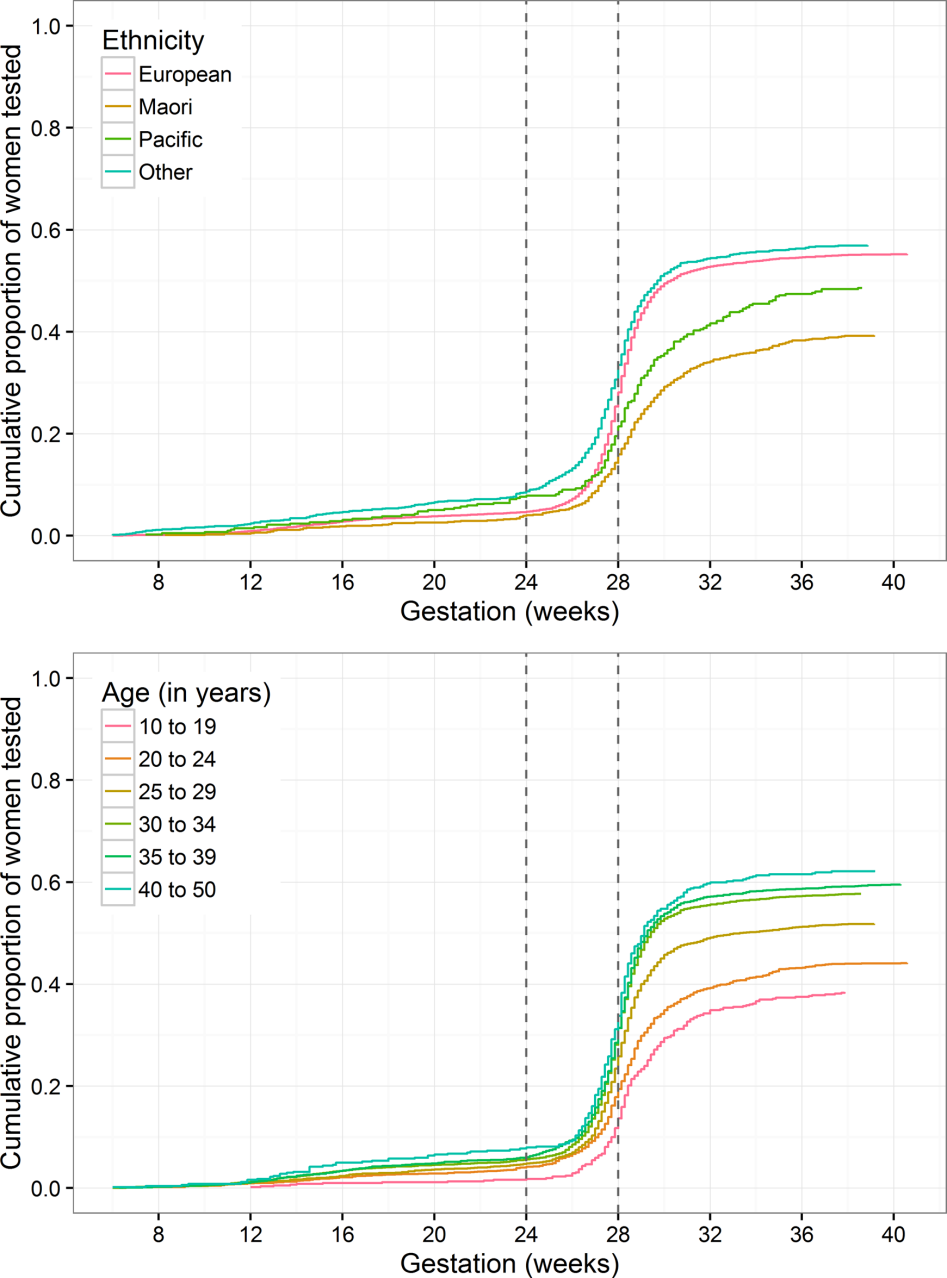
Cumulative Uptake of the Gestational Diabetes Screen (GCT or GTT) by Weeks’ Gestation from Conception Through to Delivery. Fig 2a. Grouped by maternal ethnicity. Fig 2b. Grouped by maternal age. GCT–Glucose Challenge Test. GTT–Glucose Tolerance Test.

GDM screening (by GCT and / or GTT) occurred in only 53.8% (6233 / 11580) of pregnancies, Māori women and those under 25 years of age were the least likely groups to be tested Table 1. The median (IQR) gestation at the GDM screen (gestation at the initial GCT in those who had a two-step screen, and at the GTT in those who had a one-step screen) was 28 weeks (27.0, 28.9) in European women, Māori 28.4 (27.1, 30.0), Pacific peoples 28.3 (26.9, 30.1), ‘Others’ 27.7 (26.0, 28.6). Overall 50% of initial GDM screens were performed after the recommended timing of ‘between 24 to 28 weeks gestation’ and compared with Europeans a larger proportion of Māori and Pacific People had their initial test after 32 weeks gestation, 13.6% and 15.6% respectively (Fig 2). Of the GDM screens, overall 74.6% of those performed ≥24 weeks’ gestation were two-step with an initial GCT. Non-European women were more likely than European women to undergo one-step screening (GTT only) and age was not a predictor, Māori 33.4% IPR 1.48 (1.27 to 1.74), Pacific People 38.5% 1.71 (1.41 to 2.08), ‘Others’ 39.0% 1.73 (1.54 to 1.94).

**Table 1:** Percentage of Women Screened for Gestational Diabetes (GCT or GTT), by Ethnicity and Age.

	Total women	GCT or GTT test	Unadjusted IPR	Adjusted IPR (by maternal age)
	n (%)	n (%)	(95% CI)	(95% CI)
**Ethnicity**				
European *Ref*	8957 (77.3)	4945 (55.2)	1	1
Māori	972 (8.4)	382 (39.3)	0.71 (0.66 to 0.77)	0.77 (0.71 to 0.84)
Pacific peoples	420 (3.6)	205 (48.8)	0.88 (0.80 to 0.98)	0.93 (0.84 to 1.02)
Other	1231 (10.6)	701 (56.9)	1.03 (0.98 to 1.09)	1.03 (0.98 to 1.08)
**Age in years**				
<20	622 (5.4)	239 (38.4)	0.74 (0.67 to 0.82)	
20 to 24	1746 (15.1)	771 (44.2)	0.85 (0.80 to 0.91)	
25 to 29 *Ref*	2734 (23.6)	1417 (51.8)	1	
30 to 34	3546 (30.6)	2046 (57.7)	1.11 (1.06 to 1.17)	
35 to 39	2428 (21.0)	1446 (59.6)	1.15 (1.09 to 1.21)	
40 +	504 (4.4)	314 (62.3)	1.20 (1.11 to 1.30)	
**Overall**	11580 (100)	6233 (53.8)		

GCT—50g glucose challenge test. GTT—75g glucose tolerance test. IPR—incidence proportion ratio.

Using the data above, the introduction of routine HbA1c testing with the first-antenatal bloods would increase the proportion of women screened for diabetes in pregnancy, in Europeans by 28.5% (1.5 fold increase), Māori 40.0% (2.0 fold increase), Pacific People 28.1% (1.6 fold increase), and ‘Others’ 26.7% (1.5 fold increase).

During the study period, HbA1c analysis was not a routine part of the first-antenatal blood screen and was offered as an add-on test. HbA1c tests were linked to 68.7% (7959/11580) of births. An HbA1c ≥5.9% (41 mmol/mol), the NZ cut-point for diagnosing prediabetes, was more likely in women aged 35 years or more and in non-European women Table 2. The early HbA1c result appears to have influenced the uptake of later GDM screening. Compared with women with HbA1c 5.0 to 5.8% (31 to 40 mmol/mol) the uptake of GDM screening was 8% less likely (IPR 0.92, 95% CI 0.88 to 0.96) in those with HbA1c under 5.0% (31 mmol/mol). There was no significant difference in GDM screening uptake in women with higher HbA1c 5.9 to 6.4% (41 to 47 mmol/mol), IPR 1.10 (95% CI 0.97 to 1.24) and HbA1c of 6.5% (48 mmol/mol) or above, IPR 0.78 (95% CI 0.54 to 1.12).

**Table 2:** Percentage of Women with an HbA1c Test Result ≥ 5.9% (41 mmol/mol), by Ethnicity and Age.

	Total women	HbA1c test ≥ 5.9%	Unadjusted IPR	Adjusted IPR (by maternal age)
	n (%)	n (%)	(95% CI)	(95% CI)
**Ethnicity**				
European *Ref*	6270 (78.8)	133 (2.1)	1	1
Māori	570 (7.2)	27 (4.7)	2.23 (1.49 to 3.35)	2.59 (1.71 to 3.93)
Pacific peoples	253 (3.2)	24 (9.5)	4.47 (2.94 to 6.80)	4.76 (3.10 to 7.30)
Other	866 (10.9)	54 (6.2)	2.94 (2.16 to 4.01)	2.99 (2.19 to 4.07)
**Age in years**				
<20	352 (4.4)	11 (3.1)	1.28 (0.66 to 2.45)	
20 to 24	1078 (13.5)	22 (2.0)	0.83 (0.50 to 1.39)	
25 to 29 *Ref*	1839 (23.1)	45 (2.4)	1	
30 to 34	2553 (32.1)	64 (2.5)	1.02 (0.70 to 1.50)	
35 to 39	1763 (22.2)	70 (4.0)	1.62 (1.11 to 2.36)	
40 +	374 (4.7)	26 (7.0)	2.84 (1.75 to 4.61)	
**Overall**	7959 (100)	238 (3.0)		

IPR—incidence proportion ratio

We applied our prevalence data for prediabetes (HbA1c 5.9 to 6.4% (41 to 47 mmol/mol)) and diabetes (HbA1c ≥ 6.5% (48 mmol/mol)) to the 2013 NZ national birthing data for 2013 [15], that reports 60,039 births overall, 26,229 to European, 13,488 to Māori, 6,161 to Pacific Island, and 14,161 to ‘Others’. We estimate that routine early pregnancy HbA1c measurement could potentially identify (n (estimated prevalence)) 2378 (3.96%) with prediabetes (European 525 (2.0%), Māori 580 (4.3%), Pacific People 480 (7.8%), ‘Others’ 793 (5.6%)) and 264 (0.44%) with type 2 diabetes (European 26 (0.1%), Māori 54 (0.4%), Pacific People 99 (1.6%), ‘Others’ 85 (0.6%)) per annum in NZ. The estimated number needed to screen to detect one case of prediabetes is 25, or one case of type 2 diabetes is 227.

## Discussion

We examined inter-ethnic antenatal screening practices to assess whether the NZ Ministry of Health proposal for universal HbA1c analysis with the first-antenatal bloods is likely to be accessed by those most at risk of type 2 diabetes. Uptake of the first-antenatal blood screen was reasonable (83.1% overall) with only a small difference seen by ethnicity and by age. In contrast, the uptake of the GDM screen was suboptimal across all ethnicities (53.8% overall) and occurred later than recommended, with 50% of tests occurring beyond 28 weeks gestation leaving less time for effective intervention in the event of a positive test. GDM screening was particularly low in Māori (39.3%) and compared with Europeans a larger proportion of Māori and Pacific People were tested beyond 32 weeks gestation by which point clinical intervention is less effective. Fortunately, all women are predicted to benefit from the introduction of HbA1c measurement with the first-antenatal bloods, particularly non-European women who were also most at risk of type 2 diabetes and prediabetes. In our population, routine HbA1c measurement at booking is predicted to increase access to antenatal diabetes screening 1.5–2.0 fold, the estimated number needed to screen to detect one case of prediabetes is 25, or one case of type 2 diabetes is 227. Detecting type 2 diabetes and prediabetes early in pregnancy allows more time for intervention to improve pregnancy outcomes and modulate lifestyle factors long-term, to improve the health of the mother and her offspring.

This is the first study comparing inter-ethnic differences in the proportion of women undergoing antenatal screening by test-type in a primary care setting, to establish the potential benefit of universal HbA1c testing in early pregnancy to detect unrecognised type 2 diabetes and prediabetes. The uptake of the first-antenatal bloods was similar to that reported for five other District Health Boards in NZ (87%) that account for 50% of annual NZ births [16]. Our low overall uptake of GDM screening is similar to that reported by other centres in NZ 50.6% to 56% [5,17]. Similarly, in Ireland a low uptake of GDM screening was reported in a primary care setting 52.7%, but a good uptake in secondary care 89.2% [7], while a nationwide study in the USA reports an overall uptake of 68% with a small disparity in uptake by ethnicity and by age [6]. Māori women were least likely to undergo GDM screening and this may explain why the reported national incidence of GDM in Māori is surprisingly low [2]. Although some women may still miss out on GDM screening in later pregnancy, universal HbA1c measurement in early pregnancy is an ideal opportunity to screen for type 2 diabetes and prediabetes. Historically, women with unrecognised type 2 diabetes and prediabetes were labelled as having GDM, yet they represent a group with a higher risk of adverse pregnancy and perinatal outcomes than women who develop GDM in the second half of pregnancy [4,18] and subsequently they stand to benefit the most from prompt recognition and intervention. We have previously reported that women with an HbA1c ≥ 5.9% to 6.4% (41 to 46 mmol/mol) have a high rate of GDM by IADPSG glucose criteria (74% by the third trimester) and moreover that irrespective of the GTT result these women have worse maternal and infant outcomes versus women with HbA1c ≤ 5.8% (≤ 40 mmol/mol) [3].

Strengths of our study include the population-based large cohort size and our access to laboratory data from all local laboratories. Limitations are as follows. We cannot rule out a slight underestimation of the number of women tested, for instance, women may have travelled and been tested in another province, or from an inability to match some laboratory data to births. This could happen if a laboratory incorrectly transposed the maternal national health identifier (NHI) or didn’t use an NHI at all (as occurred with a number of first-antenatal blood screens), in which case we attempted to match the test result by using maternal date of birth. Also, in a small proportion of women the early pregnancy HbA1c result influenced the uptake of the GDM screen, this was not intentional practice during the study period. Importantly, the uptake of GDM screening was 8% less likely in women with HbA1c < 5% (<31 mmol/mol) possibly due to false reassurance. This highlights the need for ongoing education for both women and caregivers alike, emphasising that the two tests are screening for two different conditions, the early pregnancy HbA1c for unrecognised type 2 diabetes and prediabetes and the GCT/GTT at 24–28 weeks gestation for pregnancy-induced diabetes (GDM) with first onset after 20 weeks gestation. Therefore a normal early pregnancy HbA1c test does not obviate the need for a GDM screen in later pregnancy, as recently reviewed [19].

We recommend that women with elevated HbA1c levels in early pregnancy are categorised as having “probable” unrecognised type 2 diabetes or prediabetes, since confirmatory testing would occur postpartum. HbA1c falls in early pregnancy due to increased red cell turnover and therefore elevated HbA1c levels at this gestation are likely true elevations. However, lifestyle intervention during pregnancy may alter glycaemic status long-term and unless the diagnosis is certain clinically a second test for confirmation is recommended [20].

The cost of an HbA1c test in our province is one third less than the cost of a GTT and it is less time consuming. Based on both our local prevalence data, and thus our number needed to screen, and the local cost of HbA1c testing we estimate that the cost per case detected of pre-diabetes is $500 NZ and of type 2 diabetes is $4540 NZ. We predict that this cost will be off-set by the early detection of type 2 diabetes and a likely reduction in pregnancy and perinatal morbidity including preeclampsia, preterm birth, operative births, and length of hospital stay for the mother and neonatal intensive care admissions. However, further study is required to quantify the cost / benefit of routine HbA1c analysis in early pregnancy.

## Conclusions

The addition of HbA1c to the first-antenatal blood tests is an ideal opportunity to identify women with undiagnosed type 2 diabetes and prediabetes in early pregnancy. Universal early pregnancy HbA1c measurement will particularly benefit populations with both high rates of unrecognised type 2 diabetes and a low uptake of GDM screening in later pregnancy. This was true for our ethnic minority groups in NZ and our results are likely to be applicable worldwide. Prompt treatment improves pregnancy outcome for women with type 2 diabetes, but further studies are required to confirm whether this is also true for women with prediabetes and whether universal early pregnancy HbA1c measurement translates to improved perinatal outcome.

## Supporting Information

S1 Table Percentage of Women with First Antenatal Bloods Taken in Christchurch, by Ethnicity and Age. (DOCX)

S2 Table Average Gestation at the First Antenatal Blood Tests, by Ethnicity and Age. (DOCX)

S1 File Minimal Data Set. (CSV)
