# Supplementary material for: Universal HbA1c Measurement in Early Pregnancy to Detect Type 2 Diabetes Reduces Ethnic Disparities in Antenatal Diabetes Screening: A Population-Based Observational Study
Source: PLoS One. 2016 Jun 7;11(6):e0156926. doi: 10.1371/journal.pone.0156926 (PMC4896429; doi:10.1371/journal.pone.0156926)
Supplement: S1 Table — (DOCX) [file pone.0156926.s001.docx]

S1 Table. Percentage of Women with First Antenatal Bloods Taken in Christchurch, by Ethnicity and Age

|  | **Total women** | **First antenatal bloods** | **Unadjusted** | **Adjusted (by maternal age)** |
| --- | --- | --- | --- | --- |
|  | **n (%)** | **n (%)** | **IPR (95% CI)** | **IPR (95% CI)** |
| **Ethnicity** |  |  |  |  |
| European *Ref* | 8957 (77.3) | 7496 (83.7) | 1 | 1 |
| Māori | 972 (8.4) | 771 (79.3) | 0.95 (0.92 to 0.98) | 0.95 (0.92 to 0.99) |
| Pacific peoples | 420 (3.6) | 323 (76.9) | 0.92 (0.87 to 0.97) | 0.92 (0.87 to 0.97) |
| Other | 1231 (10.6) | 1029 (83.6) | 1.00 (0.97 to 1.03) | 1.00 (0.97 to 1.03) |
| **Age in years** |  |  |  |  |
| <20 | 622 (5.4) | 509 (81.8) | 1.00 (0.96 to 1.05) |  |
| 20 to 24 | 1746 (15.1) | 1434 (82.1) | 1.01 (0.98 to 1.04) |  |
| 25 to 29 *Ref* | 2734 (23.6) | 2230 (81.6) | 1 |  |
| 30 to 34 | 3546 (30.6) | 2969 (83.7) | 1.03 (1.00 to 1.05) |  |
| 35 to 39 | 2428 (21.0) | 2049 (84.4) | 1.03 (1.01 to 1.06) |  |
| 40 + | 504 (4.4) | 428 (84.9) | 1.04 (1.00 to 1.08) |  |
| **Overall** | 11580 (100) | 9619 (83.1) |  |  |

IPR - incidence proportion ratio. CI - confidence intervals.
