# Supplementary material for: Universal HbA1c Measurement in Early Pregnancy to Detect Type 2 Diabetes Reduces Ethnic Disparities in Antenatal Diabetes Screening: A Population-Based Observational Study
Source: PLoS One. 2016 Jun 7;11(6):e0156926. doi: 10.1371/journal.pone.0156926 (PMC4896429; doi:10.1371/journal.pone.0156926)
Supplement: S2 Table — (DOCX) [file pone.0156926.s002.docx]

S2 Table. Average Gestation at the First Antenatal Blood tests, by Ethnicity and Age

|  | **Total women** | **Gestation of test (weeks)** | | | | |
| --- | --- | --- | --- | --- | --- | --- |
|  | **n (%)** | **Mean** | **(SD)** | **Median** | **(IQR)** | |
| **Ethnicity** |  |  |  |  |  | |
| European | 7496 (77.9) | 8.2 | (4.8) | 6.9 | (5.3 9.4) | |
| Māori | 771 (8.0) | 11.0 | (7.3) | 8.3 | (6.0 13.3) | |
| Pacific peoples | 323 (3.4) | 13.6 | (8.5) | 10.3 | (7.0 17.6) | |
| Other | 1029 (10.7) | 9.1 | (5.9) | 7.0 | (5.6 10.0) | |
| **Age in years** |  |  |  |  |  | |
| <20 | 509 (5.3) | 10.2 | (7.2) | 7.4 | (5.4 12.0) | |
| 20 to 24 | 1434 (14.9) | 9.4 | (6.6) | 6.9 | (5.3 10.6) | |
| 25 to 29 | 2230 (23.2) | 8.5 | (5.5) | 6.6 | (5.1 9.6) | |
| 30 to 34 | 2969 (30.9) | 8.3 | (4.8) | 6.9 | (5.4 9.6) | |
| 35 to 39 | 2049 (21.3) | 8.6 | (4.9) | 7.4 | (5.7 10.0) | |
| 40 + | 428 (4.4) | 9.0 | (5.0) | 7.7 | (6.0 10.4) | |
| **Overall** | 9619 (100) |  |  |  |  |  |

SD – standard deviation, IQR – interquartile range
